# European survey on preanalytical sample handling – Part 2: Practices of European laboratories on monitoring and processing haemolytic, icteric and lipemic samples. On behalf of the European Federation of Clinical Chemistry and Laboratory Medicine (EFLM) Working Group for the Preanalytical Phase (WG-PRE)

**DOI:** 10.11613/BM.2019.020705

**Published:** 2019-06-15

**Authors:** Janne Cadamuro, Giuseppe Lippi, Alexander von Meyer, Mercedes Ibarz, Edmee van Dongen, Lases, Michael Cornes, Mads Nybo, Pieter Vermeersch, Kjell Grankvist, Joao Tiago Guimaraes, Gunn B.B. Kristensen, Barbara de la Salle, Ana-Maria Simundic

**Affiliations:** 1 Department of Laboratory Medicine, Paracelsus Medical University, Salzburg, Austria; 2 Section of Clinical Chemistry, University of Verona, Verona, Italy; 3 Institute of Laboratory Medicine, Kliniken Nordoberpfalz AG and Klinikum St. Marien, Weiden and Amberg, Germany; 4 Department of Laboratory Medicine, University Hospital Arnau de Vilanova, IRBLleida, Lleida, Spain; 5 Department of Clinical Chemistry, Amsterdam UMC, University of Amsterdam, Amsterdam, The Netherlands; 6 Clinical Chemistry Department, Worcestershire Acute Hospitals NHS Trust, Worcester, UK; 7 Department of Clinical Biochemistry and Pharmacology, Odense University Hospital, Odense, Denmark; 8 Clinical Department of Laboratory Medicine, University Hospitals Leuven, Leuven, Belgium; 9 Department of Medical Biosciences, Clinical Chemistry, Umea University, Umea, Sweden; 10 Department of Clinical Pathology, São João Hospital Center, Department of Biomedicine, Faculty of Medicine, and EPI Unit, Institute of Public Health, University of Porto, Porto, Portugal; 11 Norwegian Quality Improvement of laboratory examinations (Noklus), Bergen, Norway; 12 UK NEQAS Haematology, West Hertfordshire Hospitals NHS Trust, operating UK NEQAS for Haematology and Transfusion, Watford, UK; 13 Department of Medical Laboratory Diagnostics, University Hospital Sveti Duh, Zagreb, Croatia

**Keywords:** preanalytics, standardization, survey

## Abstract

**Introduction:**

No guideline currently exists on how to detect or document haemolysis, icterus or lipemia (HIL) in blood samples, nor on subsequent use of this information. The EFLM WG-PRE has performed a survey for assessing current practices of European laboratories in HIL monitoring. This second part of two coherent articles is focused on HIL.

**Materials and methods:**

An online survey, containing 39 questions on preanalytical issues, was disseminated among EFLM member countries. Seventeen questions exclusively focused on assessment, management and follow-up actions of HIL in routine blood samples.

**Results:**

Overall, 1405 valid responses from 37 countries were received. A total of 1160 (86%) of all responders stating to analyse blood samples - monitored HIL. HIL was mostly checked in clinical chemistry samples and less frequently in those received for coagulation, therapeutic drug monitoring and serology/infectious disease testing. HIL detection by automatic HIL indices or visual inspection, along with haemolysis cut-offs definition, varied widely among responders. A quarter of responders performing automated HIL checks used internal quality controls. In haemolytic/icteric/lipemic samples, most responders (70%) only rejected HIL-sensitive parameters, whilst about 20% released all test results with general comments. Other responders did not analysed but rejected the entire sample, while some released all tests, without comments. Overall, 26% responders who monitored HIL were using this information for monitoring phlebotomy or sample transport quality.

**Conclusion:**

Strategies for monitoring and treating haemolytic, icteric or lipemic samples are quite heterogeneous in Europe. The WG-PRE will use these insights for developing and providing recommendations aimed at harmonizing strategies across Europe.

## Introduction

Laboratory results play an essential role in both medical decision-making and in patient management. In many clinical scenarios laboratory test results are essential to make the right diagnosis or choose the right treatment regime (*e.g.* HbA1c for diabetes mellitus or cardiac troponins for the diagnosis of non-ST-elevation myocardial infarction) (*1*, *2*). For this reason, laboratory test results need to be of the highest possible quality. When analysing error rates in the total testing process for improving quality, it becomes clear that focusing on the preanalytical process is inevitable, as the majority of errors occur within this phase (*3*). Looking further into these errors, haemolytic samples are described as the most burdensome and most frequent problem within the preanalytical phase (*4*, *5*). Haemolysis is predominantly the result of incorrect sample handling outside the laboratory environment with subsequent *in vitro* rupture of erythrocytes (*6*). Additionally, incorrect sample handling may lead to rupture of white blood cells and platelets with subsequent increase of intracellular substances in the plasma/serum such as potassium (*7*). Therefore, when analysing the underlying cause, actions can be taken to lower the number of haemolytic samples and then improve analytical quality (*8*).

This is quite different for lipemic and icteric samples, which are a result of endogenous/*in vivo* factors, potentially interfering with analytical methods (*9*, *10*). Lipemic samples can often be used for further analyses after applying different delipidation strategies (*11*). However, the occurrence of these factors is sometimes difficult to reduce or avoid and has to be dealt with, even if this means that certain analytes cannot be reliably measured in the respective patient.

Despite some isolated recommendations, no real consensus has been reached so far on how to measure haemolysis, icterus or lipemia (HIL) or how to use these results for interpretation and reporting of the potentially affected laboratory test results (*12*-*14*). Additionally, strategies for measuring, evaluating, avoiding and reporting HIL are quite heterogeneous between laboratories (*15*, *16*). Therefore, the European Federation of Clinical Chemistry and Laboratory Medicine (EFLM) Working Group for the Preanalytical Phase (WG-PRE) has surveyed European laboratories on preanalytical sample handling with the aim of using the data to provide recommendations and tools for harmonizing preanalytical processes. This manuscript, the second of two parts presenting the results of this survey, focuses on how laboratories across Europe measure, monitor and evaluate HIL, and how they use respective results thereof.

## Materials and methods

As described in part 1 of this series of manuscripts, a survey has been developed by the EFLM WG-PRE, inheriting 39 questions, 17 of which concerned the measurement, evaluation and follow up actions of HIL in routine blood samples (*17*). (The full survey including all questions, answer options and display conditions can be downloaded as Supplemental file of part 1 of this series). Questions were dynamically hidden or shown to participants based on answers provided to previous questions using an automated online survey tool (LimeSurvey, LimeSurvey GmbH, Hamburg, Germany).

After approval by the EFLM Scientific Committee and the EFLM Executive Board, the survey was sent to EFLM members through the European Organisation for External Quality Assurance Providers in Laboratory Medicine (EQALM) network or EFLM national societies (when an EQALM organization was unavailable in the country). Evaluation of results was performed using IBM SPSS Statistics V.24 (IBM, Armonk, New York, USA). Answers from non-EFLM member countries were not incorporated in the evaluation. In country-specific sub-analyses, countries with only five responders or less were also eliminated since these nations were insufficiently represented to display the situation in the entire country. According to the journals guideline, percentages are rounded and shown in whole numbers, except those < 10% if necessary and applicable (*18*).

## Results

Overall, 1416 participants from 45 countries completed the survey. Eleven of these responses were removed as they were provided by non-EFLM member countries, leaving 1405 responses from 37 countries. Of the remaining responders, 58 stated that they were not involved in blood sample analysis and were therefore not introduced to the questions regarding HIL. Haemolysis, icterus and/or lipemia was monitored by 1160 responders, reflecting 92% of the 1265 responders who declared to monitor/document preanalytical errors and 86% of the 1347 responders who stated to analyse blood samples. This again means that 14% (N = 187) of responders stating to analyse blood samples, declared to neither monitor preanalytical errors in general nor HIL in particular. These numbers differed throughout EU members (Table 1). The responders monitoring preanalytics, but not HIL in particular (N = 105), were mostly from public laboratories, processing only few samples or were handling mostly other samples types (*e.g.* microbiological samples) (Table 2). An 80% of these responders reported that their laboratory was accredited, certified or similar. The amount of responders not performing HIL checks decreased with number of samples processed *per* day.

**Table 1:** Number and origin of participants routinely monitoring haemolysis/icterus/lipemia

	**Total participating laboratories (N)**	**Laboratories monitoring HIL, N (%)**
Albania	14	13 (93)
Austria	56	49 (88)
Belgium	60	60 (100)
Bosnia and Herzegovina	7	6 (86)
Bulgaria	12	11 (92)
Croatia	60	60 (100)
Cyprus*	1	/
Czech Republic	60	58 (97)
Denmark	27	25 (93)
Estonia	6	6 (100)
Finland	17	15 (88)
France	192	171 (89)
Germany	51	50 (98)
Greece	7	7 (100)
Hungary	16	16 (100)
Ireland	18	18 (100)
Italy	58	55 (95)
Latvia*	1	/
Lithuania*	1	/
Luxembourg*	3	/
Macedonia	16	14 (88)
Montenegro*	4	/
Netherlands	79	65 (82)
Norway	54	44 (81)
Poland*	3	/
Portugal	57	49 (86)
Romania*	3	/
Russia	20	19 (95)
Serbia	51	50 (98)
Slovakia	11	9 (82)
Slovenia	23	22 (96)
Spain	111	106 (95)
Sweden	14	14 (100)
Switzerland	53	46 (87)
Turkey	25	24 (96)
United Kingdom (Great Britain)	72	60 (83)
Ukraine*	2	/
**Total**	**1265**	**1160 (92)**
HIL - haemolysis/icterus/lipemia. Only answers from European countries stating to monitor preanalytical errors. *Evaluation from countries with less than 6 responders was eliminated since these answers most probably did not reflect the situation in the entire country.		

**Table 2:** Basic data of participants including the number of laboratories not monitoring haemolysis/icterus/lipemia

	**Overall** **(N = 1265)**	**Not monitoring HIL** **(N = 105)**
	N (%)*	N (%)**
**Please state if you work in a:**		
Primary Care Laboratory	228 (18)	18 (7.9)
Hospital laboratory	496 (39)	53 (10.7)
Laboratory that serves both primary care and hospital (in- and outpatients)	541 (43)	34 (6.3)
**Please state the type of institution you work in:**		
Privately owned (for-profit) laboratory	371 (29)	20 (5.4)
Public (non-profit) laboratory	894 (71)	85 (9.5)
**What analytic department do you mainly work in?**		
General Clinical Chemistry	482 (38)	18 (3.7)
I work in many different analytic departments	322 (25)	11 (3.4)
Leading/Supervising position (*e.g.* head of department)	176 (14)	5 (2.8)
Haematology	65 (5.1)	11 (17)
Coagulation	12 (0.9)	1 (8.3)
Toxicology/TDM	5 (0.4)	2 (40)
Molecular biology	12 (0.9)	5 (42)
Microbiology	70 (5.5)	31 (44)
Reception/Distribution of samples	8 (0.6)	1 (12)
POCT	4 (0.3)	1 (25)
Quality Management	57 (4.5)	3 (5.3)
Transfusion	5 (0.4)	4 (80)
Clinical Pathology	1 (0.1)	1 (100)
Endocrinology	7 (0.6)	2 (29)
Serology/Virology	2 (0.2)	0 (0)
Other	12 (0.9)	4 (33)
Immunology	22 (1.7)	5 (23)
No answer	3 (0.2)	0 (0)
**Samples per day**		
< 500	572 (45)	78 (14)
500–3000	484 (38)	26 (5.4)
3001–10,000	172 (14)	0 (0)
> 10,000	37 (2.9)	1 (2.7)
**Is your laboratory accredited, certified or similar?** *(Multiple answers possible)*		
ISO 15189	582 (46)	48 (8.2)
ISO 17025	63 (5.0)	6 (9.5)
ISO 9001	239 (19)	18 (7.5)
ISO 22870	17 (1.3)	2 (12)
National standard	216 (17)	15 (6.9)
Ongoing accreditation/certification	26 (2.1)	6 (23)
Other	29 (2.3)	0 (0)
No accreditation/certification	245 (19)	21 (8.6)
TDM – therapeutic drug monitoring. POCT – point of care testing. Only answers from European countries stating to monitor preanalytical errors. *Percentage of total. **Percentage of the respective group.		

**Figure 1:**
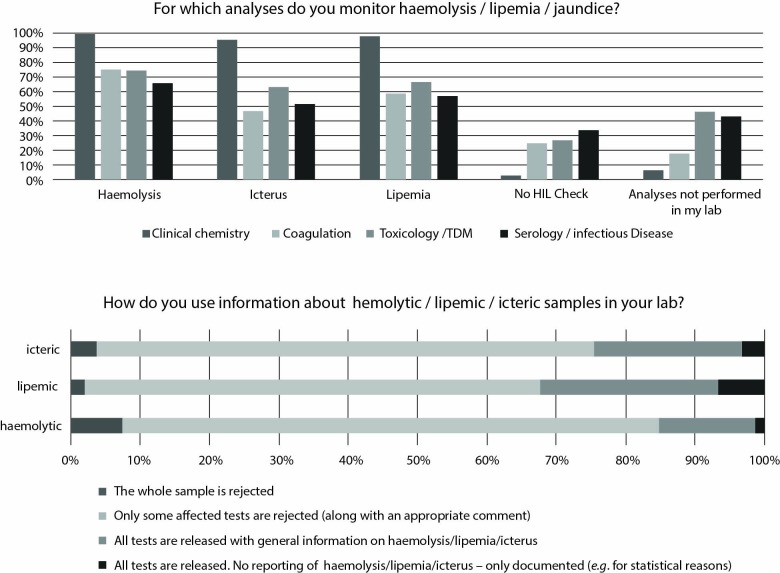
Answers to questions related to HIL monitoring and further usage

Of all responders performing HIL check, most stated to do so in samples for clinical chemistry. In samples received for coagulation, toxicology/therapeutic drug monitoring (TDM) and serology/infectious disease analyses, HIL checks were performed less frequently (Figure 1).

**Figure 2:**
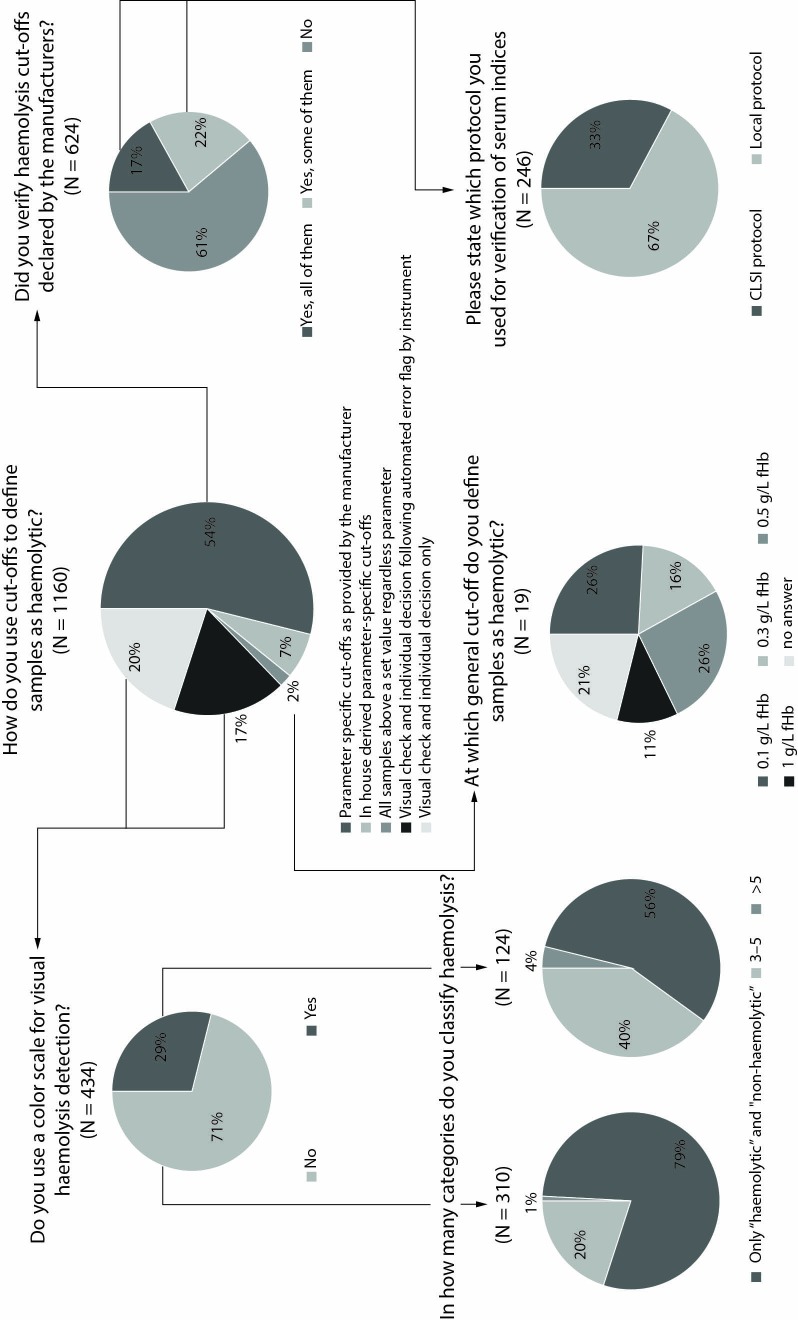
Answers to questions related to defining samples as haemolytic. CLSI - Clinical and Laboratory Standards Institute. fHb - free haemoglobin.

Measurement of HIL was performed either automatically by HIL indices, by visual inspection of samples or by a combination of automatic and visual methods by 43% (N = 493), 30% (N = 348) and 28% (N = 319) of responders, respectively. Of responders using automated HIL measurement, 25% (N = 203) stated that the quality of these measurements was regularly checked using internal quality controls (IQCs). Answers to the question how responders are using information about HIL samples for rejecting or reporting analyses are also shown in Figure 1. Most responders used analyte-specific thresholds to define samples as haemolytic, provided either by the manufacturer of the assay (54%; N = 624) or in-house derived (7.2%; N = 83) (Figure 2). Of the 624 responders committed to manufacturer´s haemolysis thresholds, 17% (N = 109) and 22% (N = 137) stated that they verified all or some of these cut-offs, respectively. This was done either according to Clinical and Laboratory Standards Institute (CLSI) recommendations (33%; N = 81) or by using local protocols (67%; N = 165) (*19*).

Cut-offs defined by responders using a universal threshold for all samples, as well as the use of colour scales in laboratories performing visual haemolysis checks, are also shown in Figure 2. Overall, 28% (N = 329) of responders monitoring HIL, declared that sample acceptance policies for samples with haemolysis, icterus or lipemia were generated in joint collaboration with clinicians. The majority of these responders (89%; N = 1034) declared not to recalculate or correct test results in haemolysed samples. Another 3% (N = 30) and 8% (N = 96) of participants stated to do so in all haemolysed samples or only when requested, respectively.

**Figure 3:**
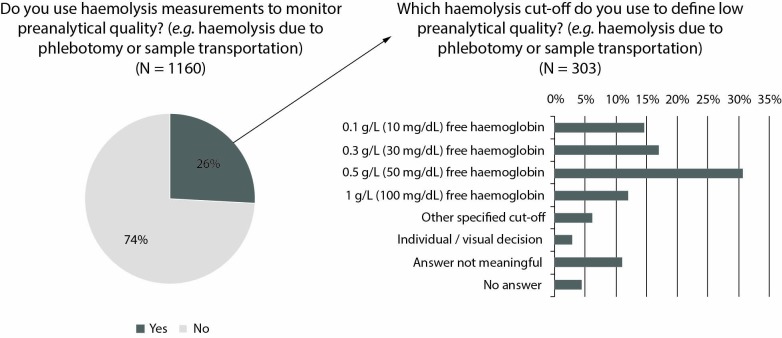
Answers related to monitoring phlebotomy quality by haemolysis index measurement

Notably, 26% (N = 303) of responders monitoring HIL also used these measurements for monitoring preanalytical quality (*e.g.* phlebotomy or transport). The cut-offs used for this aim are shown in Figure 3.

**Figure 4:**
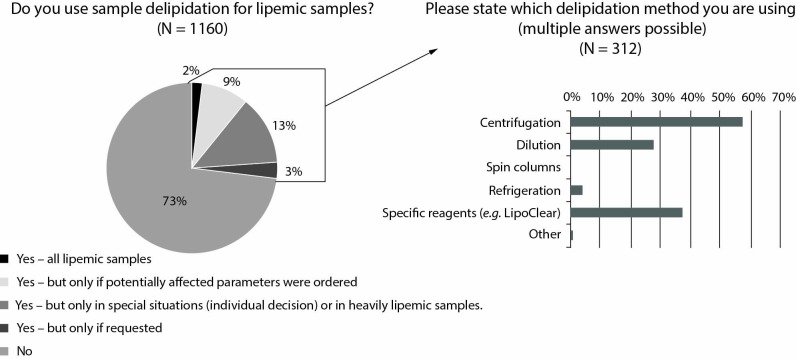
Answers related to delipidation strategies

Additional reflective measurements were always/sometimes performed by 378 (33%)/346 (30%) and 424 (37%)/282 (24%) of responders for triglycerides in lipemic samples or bilirubin in icteric samples, respectively. Information on the number of responders using delipidation techniques in lipemic samples is shown in Figure 4.

## Discussion

Haemolysis, icterus and lipemia as analytically interfering substances are still the most frequent preanalytical issues that medical laboratories have to face. Whilst haemolysis is largely preventable, as it is mostly the consequence of wrong sample handling, icterus and lipemia are patient-related endogenous effects. In either case, it is important to know if, and to what extent, any of these substances are present in the sample in order to interpret test results correctly, reject the sample or suppress the data. Since there seems to be a large heterogeneity in the field of identifying, measuring or evaluating haemolytic, icteric or lipemic samples, the WG-PRE issued a survey throughout Europe regarding this topic, with the aim of providing recommendations and tools for harmonization of these processes. We found that 86% of responders stated to analyse blood samples were monitoring HIL. The remaining 187 (14%) responders declared either not to monitor preanalytical errors in general or HIL in particular. These numbers differed slightly by country, possibly depending on type of laboratories the survey was sent out to by the national external quality assessment (EQA) provider. Those who do not monitor HIL, mostly originated from responders of small public (non-profit) laboratories, with less than 500 samples *per* day and were laboratories mainly focused on other types of samples (*e.g.* microbiology and infectious diseases, transfusion medicine). Interestingly, the amount of responders not performing HIL checks did not differ much between those accredited/certified according to the ISO 15189, the ISO 17025 or the ISO 9001 compared to those without any form of accreditation/certification. Overall, 80% (N = 84) of laboratories not performing HIL checks stated to be accredited, certified or similar, making up 19% of all laboratories analysing blood samples. This is an interesting finding, since standards like the ISO 15189 demand identification and report of HIL and other influencing substances, whenever appropriate (*20*). When only evaluating results obtained from responders stating to follow the ISO 15189 regulations, we found that 10% (N = 59) of these were not monitoring preanalytical errors in general or HIL in particular. Similar to our findings in the first part of this study, we found a clear correlation between the number of processed samples *per* day (*e.g.* the size of the laboratory) and monitoring of samples for HIL interferences (*17*). Bigger laboratories tend to perform HIL checks more than smaller ones, possibly influenced by the level of automation and/or the parameter portfolio.

When we asked for the types of analytes on which a HIL check was performed, it became evident that this was mostly done for routine clinical chemistry analyses and far less for coagulation, toxicology or serology tests. This correlates well with the amount of publications available for HIL interference within these laboratory departments. While there is much literature available on HIL in clinical chemistry, the number of research articles for others is rather limited (*21*-*24*). Additionally, automated HIL checks using serum index analyses have become very common in clinical chemistry instruments, but are only slowly emerging in the other fields.

In our survey, nearly half of the laboratories monitoring HIL did so by automatic HIL measurements. This fact is encouraging, as harmonization of these measurements is progressing, even if measurements themselves vary between instruments (*15*). However, a large amount of laboratories across Europe (58%) were still using visual inspection of sample exclusively, or additional to automatic detection of HIL, a technique which has been shown to be unreliable and plagued by high inter-observer variability (*25*, *26*). It is almost impossible to visually identify haemolysis in samples with less than 0.3 g/L of free haemoglobin (fHb), a limit where results on parameters like lactate dehydrogenase (LD), aspartate-aminotransferase (AST) or neuron specific enolase (NSE) are already significantly biased (*23*). Therefore, whenever possible, visual HIL assessment should be replaced with automatic quantitative measurement of these indices. In respective tenders, the functionality of analytical instruments for measuring serum indices should be made mandatory. If no such measurement possibility is available, a standardized colour scale would be the next best thing to use. However, according to our survey, only 29% (N = 124) of laboratories visually checking for haemolysis did so. The remaining 71% (N = 310) decided individually, mostly grading only into “haemolysed” or “non-haemolysed”. This procedure may jeopardize patient safety.

Analysis of serum indices is often not considered as an analytical parameter such as potassium or troponin. Hence, HIL checks are usually not controlled by IQCs or EQA programs. However, as these measurements are used to validate the test results of other parameters or for monitoring sample quality in general, they are directly influencing test results on laboratory reports. Consequently, HIL measurements should be included in any quality management system, monitoring their quality internally on a daily basis, as well as externally, by participating in respective EQA schemes. In our survey, we found that only a quarter of responders (N = 203) performing any form of automatic HIL check were using an IQC to monitor these analyses. However, at the time this survey was issued, commercial IQC was unavailable for HIL measurements, nor was there any guideline for preparing respective in-house IQCs (*27*). On the contrary, EQA programs to assess HIL measurements were available but, as shown in Part 1 of these twin manuscripts, only a small amount of laboratories were using them. Interestingly, another 29% (N = 396) of participants stated not to be interested in participating is such an EQA program (*17*).

After measuring HIL, results must be interpreted for the respective sample. Hence, laboratories need to have a HIL cut-off that defines the threshold above which an analyte is analytically biased. When asked specifically for haemolysis interference, we found that over 60% of the responders performing HIL checks were using parameter-specific cut-offs for this interpretation, of which the vast majority were declared by the manufacturer. However, nearly two thirds of these laboratories did not verify them. Of those who claimed to do so, 67% used a local protocol instead of the official CLSI guideline (*19*). When adopting manufacturers HIL cut-offs, laboratories should be aware that manufacturers of *in vitro* diagnostic (IVD) analytical systems do not often fully adhere to respective CLSI guidelines on interference testing. Therefore, we highly recommend verifying these results for those parameters known to be affected by haemolysis, icterus or lipemia. For example, manufacturers may use a general 10% deviation as the allowed analytical bias for defining cut-offs for each parameter without considering individual intra- and inter-assay, biologic variability as well as clinical relevance. Additionally, often data on how cut-offs were calculated by manufacturers are lacking, which is the reason why the WG-PRE recently called for more transparency in manufacturers declarations on serum indices (*28*). Without the support of manufacturing companies, single laboratories will not be able to establish reliable HIL cut-offs, neither financially nor in terms of human resources.

A small proportion of participating laboratories are using one solitary H-cut-off, independent from the analytes ordered in the respective sample, to decide whether or not to reject the sample. The exact cut-off value in use varied greatly from 0.1 to 1 g/L of fHb. As haemolysis measurement is known to differ between analytical platforms, this finding, at least in part, may also be influenced by the analytical instrument in use (*15*). Every sample a laboratory receives is haemolytic to some degree. The levels are mostly so low that they are barely detectable and do not interfere with analytical testing. Nevertheless, it is important to define a threshold above which test results are significantly biased. This should be carried out for each laboratory parameter individually, *e.g.* following the CLSI guideline on interference testing (*19*).

After defining a sample as haemolytic for further analyses, actions must be taken, and the interference needs to be reported to the requesting clinician. Most of the responders to our survey stated to reject only those parameters affected by haemolysis (77%), icterus (66%) or lipemia (72%), accompanied by an appropriate comment on the report. Another 14%/26%/21% choose to release all test results with a general information on haemolysis/icterus/lipemia. A quite high proportion of laboratories rejected the entire sample when haemolysed (7.4%), icteric (3.8%) or lipemic (2.1%). Additionally, a non-negligible amount of laboratories released all tests, without including any comment on the report as to whether the sample was haemolytic (1.2%), icteric (3.3%) or lipemic (6.6%). Rejecting samples which at least in part would have been measurable, potentially harms patients in the same way as releasing test results clearly biased by HIL interference without appropriate commenting. Both of which may lead to wrong, missed or delayed diagnosis of the patient, one of the most burdensome medical errors (*29*).

Overall, procedures on how to act upon haemolytic, icteric or lipemic samples are very heterogeneous. The reason may be the lack of available recommendations or guidelines. The WG-PRE therefore recently published such a recommendation, which divides test result deviations due to haemolysis into analytically and clinically significant, including a proposal on how to report these results (*14*). Reporting results which are biased above the clinically relevant cut-off with or without any comment should be avoided (*30*).

Another possible use of systematic haemolysis measurements in all samples may be monitoring of sample quality in terms of phlebotomy practices. For example, intravenous (IV) catheter blood collections or the use of high vacuum tubes may lead to higher haemolysis rates (*31*, *32*). Therefore, many publications have used the haemolysis index to demonstrate the effect of phlebotomy improvement interventions (*e.g.* educational or the change of phlebotomy equipment) (*33*-*35*). This principle could be used by laboratories to monitor phlebotomy quality in their health care setting as quality indicator, then taking further actions when the situation worsens *e.g.* on a specific ward (*36*). Tools to document and evaluate haemolysis indices are freely available (*37*, *38*). However, care has to be taken as haemolysis may also originate *in vivo* as a severe symptom of an underlying disease of the patient (*39*). In our survey, only 26% (N = 303) of responders who were performing HIL checks were actually using haemolytic information to monitor phlebotomy quality. Of those, 31% (N = 93) used a cut-off of 0.5 g/L fHb, which mirrors that recommended by the International Federation of Clinical Chemistry and Laboratory Medicine (IFCC) (*40*). The remaining 69% of responders were using very different cut-offs, individual decisions, visual checks, gave no answer or did not understand the question properly. As mentioned above, haemolysis measurements differ between analytical platforms, which in turn may influence the cut-off used for deciding on phlebotomy quality. Nevertheless, we highly recommend documenting information on haemolysis measurements as quality indicator, using the recommended cut-off as well as the above-mentioned tools.

Unlike haemolytic samples, which may be avoided by improving phlebotomy or transportation processes, icterus and lipemia are far less preventable since they are *in vivo* interfering substances. We found that only 27% (N = 312) of responders measuring lipemia in their samples used any kind of delipidation methods such as centrifugation, dilution or specific reagents, in order to clear the plasma prior to analyses. These methods, as well as the strategies to define which sample should undergo delipidation and when to measure triglycerides or bilirubin in lipemic/icteric samples, seem very heterogeneous, mostly based on individual decision.

As limiting factors to our survey, we want to mention that, although we advised laboratories to give only one answer *per* facility, we cannot completely rule out multiple answers from the same facility in some cases. Due to data protection regulations, we refrained from collecting the exact IP addresses of responders. Additionally, we are aware that some countries are overrepresented (*e.g.* France, Spain), whilst others might be underrepresented. We tackled this issue by providing country-specific evaluation wherever appropriate.

In conclusion, we found that haemolysis, icterus and lipemia are measured by most responders of our survey across Europe, especially in samples for clinical chemistry analyses. Most participants stated to use parameter-specific HIL cut-offs, however, mostly without prior verification. The process on how to deal with haemolytic/lipemic/icteric samples and with test results, which might be affected, seems very heterogeneous. In striving for optimal quality of laboratory values, harmonization and standardization of pre- and postanalytical processes is needed. With the results from this European survey, the WG-PRE now has the necessary basis to develop and provide specific guidelines and recommendations in order to achieve this ambitious goal.
